# Isoforms of the neuropeptide myosuppressin differentially modulate the cardiac neuromuscular system of the American lobster, *Homarus americanus*

**DOI:** 10.1152/jn.00338.2021

**Published:** 2022-01-19

**Authors:** Emily R. Oleisky, Meredith E. Stanhope, J. Joe Hull, Patsy S. Dickinson

**Affiliations:** ^1^Department of Biology, Bowdoin College, Brunswick, Maine; ^2^United States Department of Agriculture-Agricultural Research Service (USDA-ARS), Maricopa, Arizona

**Keywords:** central pattern generator, cardiac ganglion, myosuppressin, neuromodulator, Homarus americanus

## Abstract

Post-translational modifications (PTMs) diversify peptide structure and allow for greater flexibility within signaling networks. The cardiac neuromuscular system of the American lobster, *Homarus americanus*, is made up of a central pattern generator, the cardiac ganglion (CG), and peripheral cardiac muscle. Together, these components produce flexible output in response to peptidergic modulation. Here, we examined the role of PTMs in determining the effects of a cardioactive neuropeptide, myosuppressin (pQDLDHVFLRFamide), on the whole heart, the neuromuscular junction/muscle, the isolated CG, and the neurons of the CG. Mature myosuppressin and noncyclized myosuppressin (QDLDHVFLRFamide) elicited similar and significant changes in whole heart contraction amplitude and frequency, stimulated muscle contraction amplitude and the bursting pattern of the intact and ligatured neurons of the ganglion. In the whole heart, nonamidated myosuppressin (pQDLDHVFLRFG) elicited only a small decrease in frequency and amplitude. In the absence of motor neuron input, nonamidated myosuppressin did not cause any significant changes in the amplitude of stimulated contractions. In the intact CG, nonamidated myosuppressin elicited a small but significant decrease in burst duration. Further analysis revealed a correlation between the extent of modulation elicited by nonamidated myosuppressin in the whole heart and the isolated, intact CG. When the neurons of the CG were physically decoupled, nonamidated myosuppressin elicited highly variable responses. Taken together, these data suggest that amidation, but not cyclization, is critical in enabling this peptide to exert its effects on the cardiac neuromuscular system.

**NEW & NOTEWORTHY** Myosuppressin (pQDLDHVFLRFamide), a well-characterized crustacean neuropeptide, and its noncyclized (QDLDHVFLRFamide) and nonamidated (pQDLDHVFLRFG) isoforms alter the output of the cardiac neuromuscular system of the American lobster, *Homarus americanus.* Mature myosuppressin and noncyclized myosuppressin elicited similar and significant changes across all levels of the isolated system, whereas responses to nonamidated myosuppressin were significantly different from other isoforms and were highly variable. These data support the diversity of peptide action as a function of peptide structure.

## Graphical abstract

**Figure:**
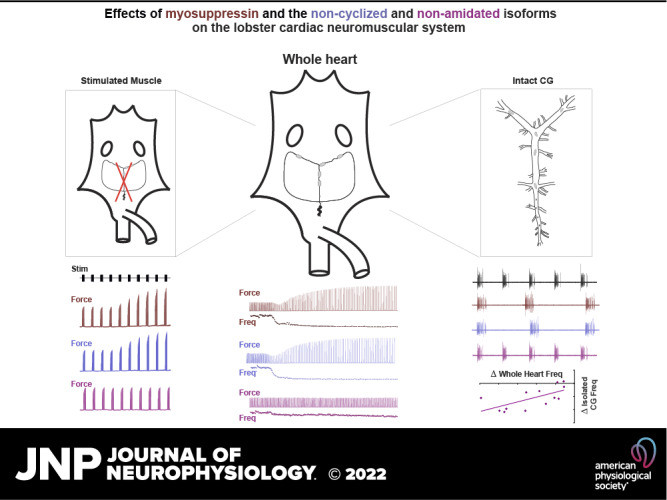

## INTRODUCTION

Central pattern generators (CPGs) are neural networks that produce rhythmic electrical outputs in the absence of sensory or central input. Circulating and locally released neuromodulators allow for flexibility in the neuronal output that underlies a variety of patterned movements such as chewing and walking (1, 2). Although various chemical molecules can serve as neuromodulators, peptides are the most expansive and diverse group (3–5).

Following enzymatic cleavage within the neurosecretory pathway, peptides undergo a variety of post-translational modifications (PTMs), including carboxy (C)-terminal amidation, cyclization of N-terminal glutamine/glutamic acid (Q/E) residues, disulfide bridging between cysteines, and sulfation of tyrosines (5). PTMs are important mechanisms that diversify peptide function and increase the functional flexibility of complex networks (6). These structural alterations regulate the interactions of molecules, maintain the stability of peptides, and play a significant role in determining the effects elicited on signaling systems.

**Figure 1.**
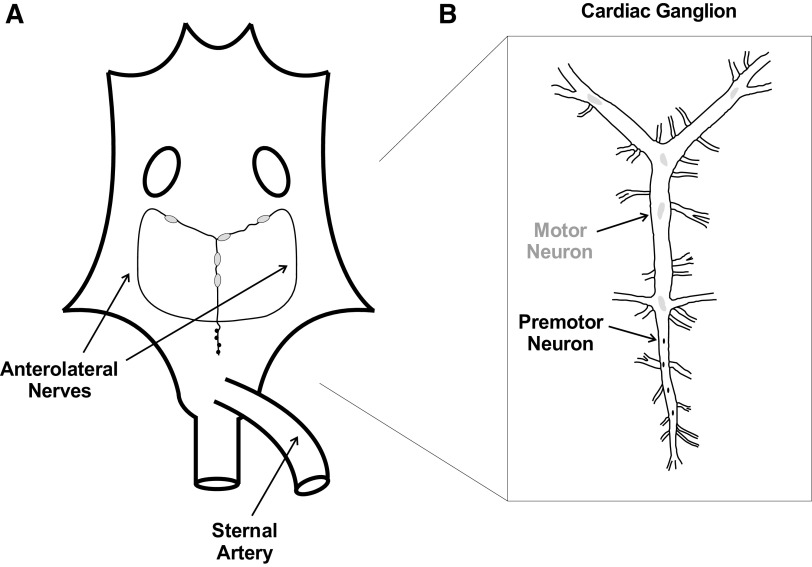
Schematic diagram of the semi-intact cardiac neuromuscular system of the American lobster, *Homarus americanus*. *A*: organization of the whole heart, depicting the location of the two anterior ostia, the cardiac ganglion with its nine neuronal cell bodies, and the posterior arteries. *B*: anatomy of the isolated cardiac ganglion. Five larger, motor neurons located in the anterolateral nerves of the ganglion are electrically and chemically coupled to the four smaller, premotor neurons located in the posterior trunk.

The cardiac neuromuscular system of the American lobster, *Homarus americanus*, serves as a model for understanding the control of rhythmic behavior (7). This CPG-effector system controls the rhythmic beating of the lobster heart via the action of the cardiac ganglion (CG) and the surrounding heart musculature (Fig. 1). The CG is composed of only nine neurons: five large, anterior motor neurons and four small, posterior premotor neurons. These premotor neurons have been viewed as the drivers of rhythmic CG activity, via synapses with motor cells that promote bursting activity. Motor neurons send feedback to the premotor neurons and endogenous driver potentials regulate the collective activity of the CG, including output frequency (8, 9). The premotor and motor neurons are both electrically and chemically coupled, and produce resilient, in-phase patterned output (10). The joint action of these neurons produces individual action potentials, or spikes, that coalesce into electrical bursts. Motor neuron output stimulates excitatory junction potentials (EJPs), which sum to contract the muscle fibers and drive heart contractions (7). The force of each contraction is thus dependent on the cycle frequency of the CG (7), as well as the duration of CG motor neuron bursts. Contraction force is, in fact, a complex function of CG frequency and duty cycle, acting via the neuromuscular transform (11).

Modulation at multiple sites within the cardiac neuromuscular system, including at the level of the neurons (i.e., the CPG) and the musculature (i.e., the periphery) has been shown in previous studies (12–18). Stevens et al. (17) demonstrated that myosuppressin (pQDLDHVFLRFamide), a highly conserved decapod neuropeptide (19), was able to decrease heart contraction frequency and elicit an initial decrease in contraction amplitude followed by a large increase in the whole heart at 10^−7^ M concentrations. Based on the neuromuscular transform (11), it is expected that a decrease in burst frequency of the CG and, thus, in contraction frequency of the whole heart, would result in decreased contraction amplitude. Indeed, Stevens et al. (17) showed, and we confirmed here, that in the isolated, intact CG, myosuppressin (10^−7^ M) elicited a decrease in burst frequency, as well as an increase in burst duration. This increase in burst duration, via the neuromuscular transform, would be predicted to result in increased contraction amplitude. To further characterize its effects, myosuppressin (10^−7^ M) was applied to semi-intact hearts driven by electrical stimuli mimicking a consistent CG bursting pattern. Even in the absence of changes in burst duration, myosuppressin caused an increase in contraction amplitude, suggesting that this component of the myosuppressin response is mediated peripherally, as well as centrally. More recently, Oleisky et al. (20) demonstrated that the premotor and motor neurons of the lobster CG are independently modulated by myosuppressin following decoupling by a physical ligature.

In a mass spectrometric study of pooled *H. americanus* nervous tissue, both full myosuppressin and an isoform lacking the N-terminal cyclization (QDLDHVFLRFamide) were identified (21). Given the possibility that the presence of multiple isoforms can increase network flexibility (22), we investigated the role played by the PTMs of myosuppressin, and how modulation of the *Homarus* cardiac neuromuscular system differed based on the PTMs of the peptide. The nonamidated isoform of myosuppressin has not, however, been seen in mass spectrometric studies of the lobster nervous system, suggesting that this peptide isoform may not contribute to peptide diversity; instead, this PTM may be required for biological activity of this peptide.

To test each PTM of myosuppressin independently, isoforms of myosuppressin lacking the N-terminal cyclization (i.e., QDLDHVFLRFamide) and C-terminal amidation (i.e., pQDLDHVFLRFG) were synthesized for comparison to the mature, fully-modified isoform (pQDLDHVFLRFamide). Given the ability of PTMs to regulate the bioactivity of peptides, we asked whether isoforms lacking each of the two known PTMs would retain bioactivity, and if so, whether this would alter the peptide’s modulatory effects at the global (i.e., whole heart), central (i.e., neural), and peripheral (i.e., neuromuscular junction) levels of the system.

## MATERIALS AND METHODS

### Animals

Adult American lobsters (*H. americanus*) were purchased from local seafood retailers (Bath & Brunswick, ME) and housed in recirculating natural seawater tanks at 10°C–12°C with ∼12-h/12-h light-dark cycles. Lobsters were fed a diet of chopped shrimp or squid once a week.

Lobsters were cold-anesthetized on ice for ∼30 min before isolation of the heart from the dorsal thoracic carapace via manual microdissection in chilled (8°C–10°C) physiological saline [composition in mmol/L: 479.12 NaCl, 12.74 KCl, 13.67 CaCl_2_, 20.00 MgSO_4_, 3.91 Na_2_SO_4_, 11.45 Trizma base, and 4.82 maleic acid (pH = 7.45); 22].

### Peptides

Myosuppressin peptides (pQDLDHVFLRFamide, QDLDHVFLRFamide, and pQDLDHVFLRFG), custom synthesized by GenScript (Piscataway, NJ), were dissolved in dimethyl sulfoxide (DMSO), due to their low solubility, and then diluted with deionized water to yield 10^−3^ M myosuppressin, 15% DMSO stock solutions. At the concentration of DMSO in the solutions here, DMSO had no effect on the endogenous physiology of the cardiac neuromuscular system (23). Aliquots of the 10^−3^ M peptide solutions were stored at −25°C and thawed and diluted in saline to 10^−6^ M or 10^−7^ M immediately before use.

### Physiology

#### Recordings.

For whole hearts, the sternal artery was cannulated and continuously perfused with cool (10°C–13°C) physiological saline at ∼2.5 mL/min. A second perfusion line was directed over the top of the heart to help maintain temperature. To record heart contractions, the anterior arteries were tied off using 6-0 Suture Silk and attached to an FT03 force transducer (Grass Natus Technologies, Pleasanton, CA) at an angle of ∼45° from the horizontal plane. To model endogenous conditions, hearts were stretched to a baseline tension of ∼2 g. After 60 min stabilization, all three myosuppressin isoforms were bath applied to each heart via the cannulated sternal artery (10^−6^ M, 15 min peptide application) in a randomized order, interspersed by 60 min saline washes.

A stimulated muscle preparation was used to test the effects of peptides on the periphery of the system, as previously described in Stevens et al. (17). In brief, the heart was cannulated and attached to a force transducer as in whole heart experiments. To eliminate spontaneous activity, the motor neuron cell bodies of the cardiac ganglion were removed through a horizontal incision anterior to the cannulated artery. A suction electrode was attached to one anterolateral nerve and used to trigger action potentials in the motor nerve. Impulses (0.5 ms, 60 Hz, 3–9 V) were delivered in 300-ms bursts, repeated at a rate of 30 bursts/min. To ensure that the heart would continue to contract for the duration of the experiment, bouts of bursts were delivered once every 90 s. Each bout consisted of 15 bursts. Spike2 v7.16 was used to control electrical impulses, which were then generated using a model 2100 A-M Systems isolated pulse simulator (A-M Systems, Carlsborg, WA) and delivered via the suction electrode. Contractions triggered by stimulation of the motor nerve were measured in control saline and in the presence of 10^−6^ M and 10^−7^ M myosuppressin.

To examine the action of myosuppressin isoforms on the isolated CG, the heart was opened along the ventral wall and the ganglionic trunk and anterolateral nerves were dissected from the surrounding musculature. To record from the intact CG, petroleum jelly wells were built around small portions of the anterolateral nerves. Bipolar stainless-steel electrodes were used for extracellular recordings, with one electrode in the well and the other in the nearby bath. To record from the physically decoupled premotor and motor neurons, the ganglion was ligatured with a single fiber of suture silk, following a previously established protocol. An additional well around the trunk of the ganglion was used to monitor premotor neuron output as previously described (20).

Physiological saline was perfused through in vitro heart preparations and superfused over isolated cardiac ganglia using a Rabbit peristaltic pump (Gilson, Middleton, WI). Temperature was maintained throughout recordings using an in-line Peltier temperature regulator (CL-100 bipolar temperature controller and SC-20 solution heater/cooler; Warner Instruments, Hamden, CT) and was monitored using a temperature probe (Warner Instruments). The output of the force transducer used for whole heart and stimulated heart preparations was amplified using an ETH-250 Bridge/Bio Amplifier. Neuronal output from isolated ganglia was amplified using a 1700 A-M Systems Differential AC amplifier (Sequim, WA) and a 440 Brownlee Precision Instrumentation amplifier (NeuroPhase, Palo Alto, CA). All data were digitized with a CED Micro 1401 digitizer and recorded using Spike2 7.17 (Cambridge Electronic Design, Cambridge, UK), with a sampling rate of 10 kHz.

### Data Analysis

Physiological recordings were analyzed with Spike2, v. 7.17 using built-in analysis features and scripts from the STG Laboratory at NJIT Rutgers.

For whole heart recordings, amplitude and frequency of contractions were averaged over 200-s intervals, with control values taken just before peptide was introduced to the bath and peptide values taken at the peak of peptide effect, near the end of the peptide application. Peak response to myosuppressin was defined as the lowest frequency and the largest amplitude contractions.

In stimulated preparations, data were averaged over three bouts of stimuli for control and peak peptide response, with control values taken shortly before addition of the peptide to the bath. Because contractions amplitude increased within each bout of stimuli, we measured only the last two contractions in each bout (i.e., the 14th and 15th stimulus-evoked contractions).

In isolated, intact ganglion preparations, ganglionic bursts were averaged over 200 s, with the control values taken just before peptide application and peptide values taken at peak response. A burst was defined as a trail of ≥5 action potentials occurring at a frequency of at least 100 Hz (19). Burst duration, burst frequency, and duty cycle were quantified. In the ligatured CG, data were averaged over 10 bursts based on previous protocol (20), and burst duration was quantified separately for premotor and motor neurons.

All statistical analyses and graphing were conducted using Prism v7.0 (GraphPad Software, San Diego, CA). To standardize for variation in baseline parameters, values are presented as percent change from baseline; only preparations that returned to baseline during saline wash were included in analysis. One sample *t* tests were used to determine if the percent change from baseline was significantly different from a hypothetical value of zero. To compare myosuppressin isoforms, one-way ANOVAs were used with Tukey multiple comparisons tests. Correlation plots from dual whole heart (*x*-axis) and isolated ganglion preparations (*y*-axis) were generated and Pearson *R* and linear regression analyses quantified the extent of correlation. *P* values of < 0.05 were significant.

### Receptor Expression

To examine myosuppressin receptor expression in the *H. americanus* cardiac muscle (CM), total RNAs from the cardiac muscle (*n* = 8 samples) were prepared as described previously (24). RNA metrics were assessed on an Agilent 2100 Bioanalyzer (Agilent Technologies, Santa Clara, CA) and cDNAs synthesized using a SuperScript III First-Strand Synthesis System (Thermo Fisher Scientific, Carlsbad, CA) with custom pentadecamers (IDT, San Diego, CA) and ∼500 ng of DNase I-treated total RNA. Fragments of HaMSRs- I-V (MT068477–MT068483) were amplified using SapphireAmp Fast PCR Master Mix (Takara Bio USA, Inc., Mountain View, CA) and gene specific primers (Table 1). To verify the integrity of the cDNA templates, a 500-bp fragment of *H. americanus* glyceraldehyde-3-phosphate dehydrogenase (GAPDH; accession no. FE043664) was likewise amplified with gene-specific primers (Table 1). PCR was performed in a 20-μL reaction volume using 0.5 μL cDNA across 8 biological replicates. Thermocycler conditions consisted of 95°C for 2 min, followed by 35 cycles of 95°C for 20 s, 56°C for 20 s, and 72°C for 30 s, with a final extension at 72°C for 5 min. The resulting products were separated on 1.5% agarose gels and visualized with SYBR Safe (Thermo Fisher Scientific). A subset of the reactions was subcloned into pCR2.1-TOPO TA (Thermo Fisher Scientific) and sequence-validated with either the Arizona State University DNA Core Laboratory (Tempe, AZ) or Retrogen Inc (San Diego, CA). Gel images were obtained on an Azure 200 Gel Imaging Workstation (Azure Biosystems, Dublin, CA) and processed for publication in Adobe Photoshop v21.2.12 (Adobe Systems Inc., San Jose, CA).

**Table 1.** Gene-specific oligonucleotide primers used

Primer	Sequence (5′->3′)
HaGAPDH 96 F	TCGGTCGTCTTGTCCTTC
HaGAPDH 599 R	CAGTGACGGCATGAACAG
HaMSR-I 653 F	ACTTCACCATCAGCACGA
HaMSR-I 1173 R	GCGGATAGATAGCACCGA
HaMSR-II 179 F	CCACCACACAAGACTCCT
HaMSR-II 682 R	GGATGTTGCAGATGACGG
HaMSR-III 350 F	CCGTGATCTGCAACATCC
HaMSR-III 816 R	CAGCAGGAAGTTGATGGC
HaMSR-IV 252 F	CTTGGCGCTGATGATCTG
HaMSR-IV 755 R	TGTAGCAGGCCATTCGAT
HaMSR-V 695 F	TGTCCAACGATGACGGAT
HaMSR-V 1229 R	TTGATGAGCGCCAACAAG

## RESULTS

### Post-Translational Modifications Alter the Modulatory Capacity of Myosuppressin

**Figure 2.**
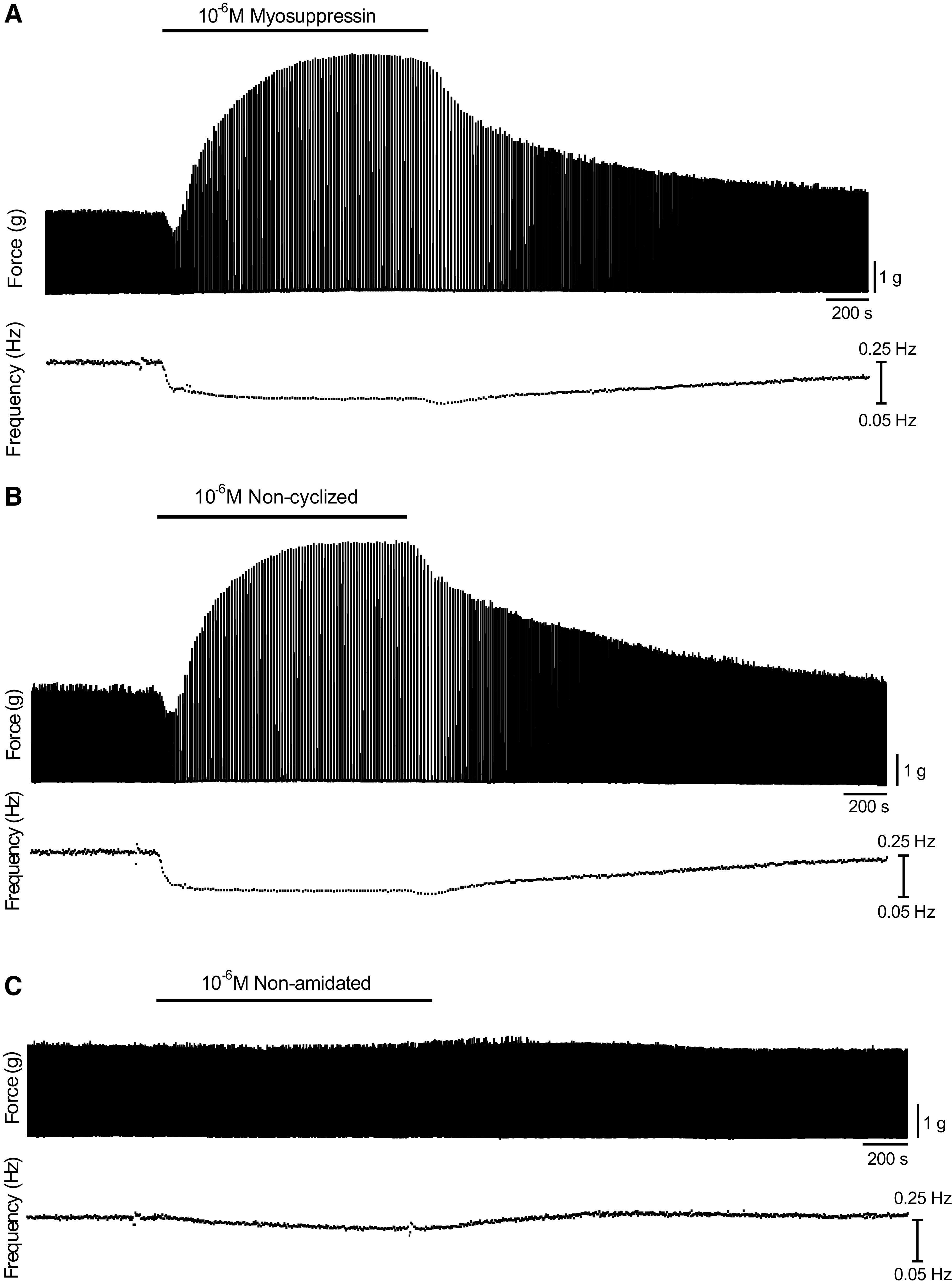
Myosuppressin isoforms altered the patterned beating of the whole heart. At 10^−6^ M, application of full myosuppressin (*A*), noncyclized myosuppressin (*B*), and nonamidated myosuppressin (*C*) qualitatively altered the pattern of whole heart contractions. Traces shown are representative of the whole heart effects across individuals.

**Figure 3.**
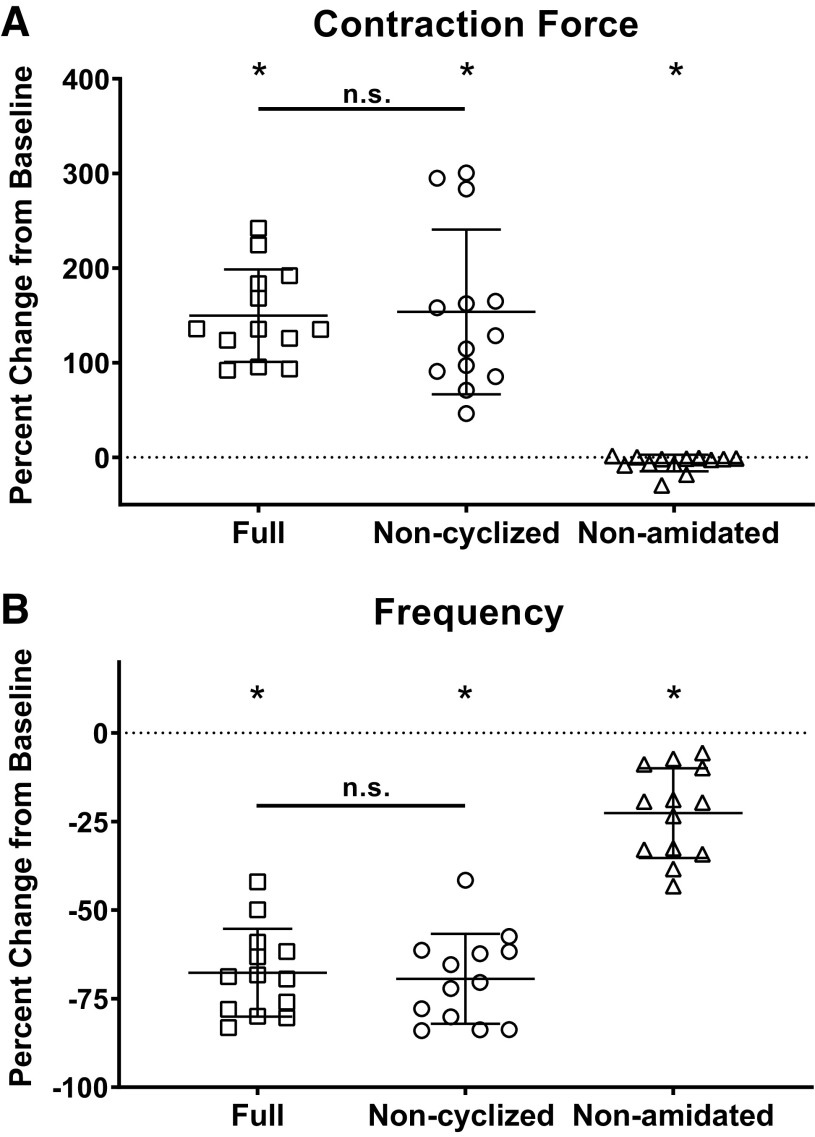
Myosuppressin and noncyclized myosuppressin exerted similar effects on the force and frequency of cardiac contractions. Pooled data from preparations (*n* = 13) exposed to 10^−6^ M concentrations of myosuppressin demonstrated that all isoforms were capable of eliciting significant changes in force (*A*) and frequency (*B*) of contraction. In both parameters measured, each isoform of myosuppressin had a significant effect on the parameter measured (one-way ANOVA, *P* < 0.0001, *n* = 13). Likewise, in all parameters, the responses to myosuppressin and noncyclized myosuppressin did not differ significantly from each other (Tukey’s multiple comparisons, *P* > 0.05, *n* = 13), but did differ significantly from the response to the nonamidated isoform (Tukey’s multiple comparisons, *P* < 0.05 for both isoforms, *n* = 13). For all parameters tested, all three isoforms caused a significant change from zero percent (one sample *t* test, *P* < 0.05 for all isoforms, *n* = 13). *Significant difference from 0% (calculated as % change from baseline), one sample *t* test, *P* < 0.05, *n* = 13, error bars represent SD.

When perfused through the whole heart, 10^−6^ M myosuppressin (pQDLDHVFLRFamide) elicits a decrease in contraction frequency and a biphasic modulation of contraction amplitude (17). Because fully modified myosuppressin (“mature myosuppressin”) includes two PTMs, an N-terminal cyclization of the pyroglutamate residue and a C-terminal amidation, and recent work has demonstrated that the presence of a myosuppressin isoform lacking at least one of these PTMs (i.e., the cyclized pyroglutamate residue) in *Homarus* neural tissues (19), we asked what role these PTMs played in determining the effect of myosuppressin on the cardiac neuromuscular system. To answer this question, we first confirmed the effects of mature myosuppressin and then perfused myosuppressin lacking the N-terminal cyclization (noncyclized myosuppressin) and myosuppressin lacking the C-terminal amidation (nonamidated myosuppressin) through the whole heart (Fig. 2). Noncyclized myosuppressin (QDLDHVFLRFamide) elicited the same effects on frequency and amplitude as mature myosuppressin, despite differential modification (Fig. 3, *A* and *B*; Tukey’s multiple comparisons, *P* > 0.05, *n* = 13). However, synthetic nonamidated myosuppressin (pQDLDHVFLRFG) consistently caused a small but significant decrease in contraction amplitude (5.9 ± 8.8% SD; Fig. 3*A*, one sample *t* test, *P* = 0.032, *n* = 13), as well as a small decrease in cycle frequency (22.6 ± 12.7% SD; Fig. 3*B*, one sample *t* test, *P* < 0.001, *n* = 13). However, unlike mature myosuppressin, the nonamidated myosuppressin did not cause an increase in contraction amplitude at any point during the peptide application, suggesting altered bioactivity due to the lack of C-terminal amidation.

The neuromuscular transform (11) suggests that the decrease in contraction frequency elicited by all three peptides results in the initial decrease in contraction amplitude observed here. Specifically, as long as the duration of bursts in the cardiac ganglion remains constant, decreases in CG burst frequency in the neurogenic lobster heart lead to decreases in contraction amplitude of the cardiac muscle. The subsequent increase in contraction amplitude recorded in response to myosuppressin may be partly explained by the neuromuscular transform, as we expect the delayed increase in burst duration in the CG elicited by myosuppressin to result in an increase in contraction amplitude. However, Stevens et al. (17) showed that the delayed increase is partially mediated at the periphery of the system. Given that PTMs affected the modulatory effects of myosuppressin on the intact cardiac neuromuscular system, we next asked whether this response was mediated by differential modulations at the peripheral (neuromuscular junction/muscle) and central (neural) level.

### Two Myosuppressin Isoforms Modulate Contractions in the Stimulated Cardiac Muscle

**Figure 4.**
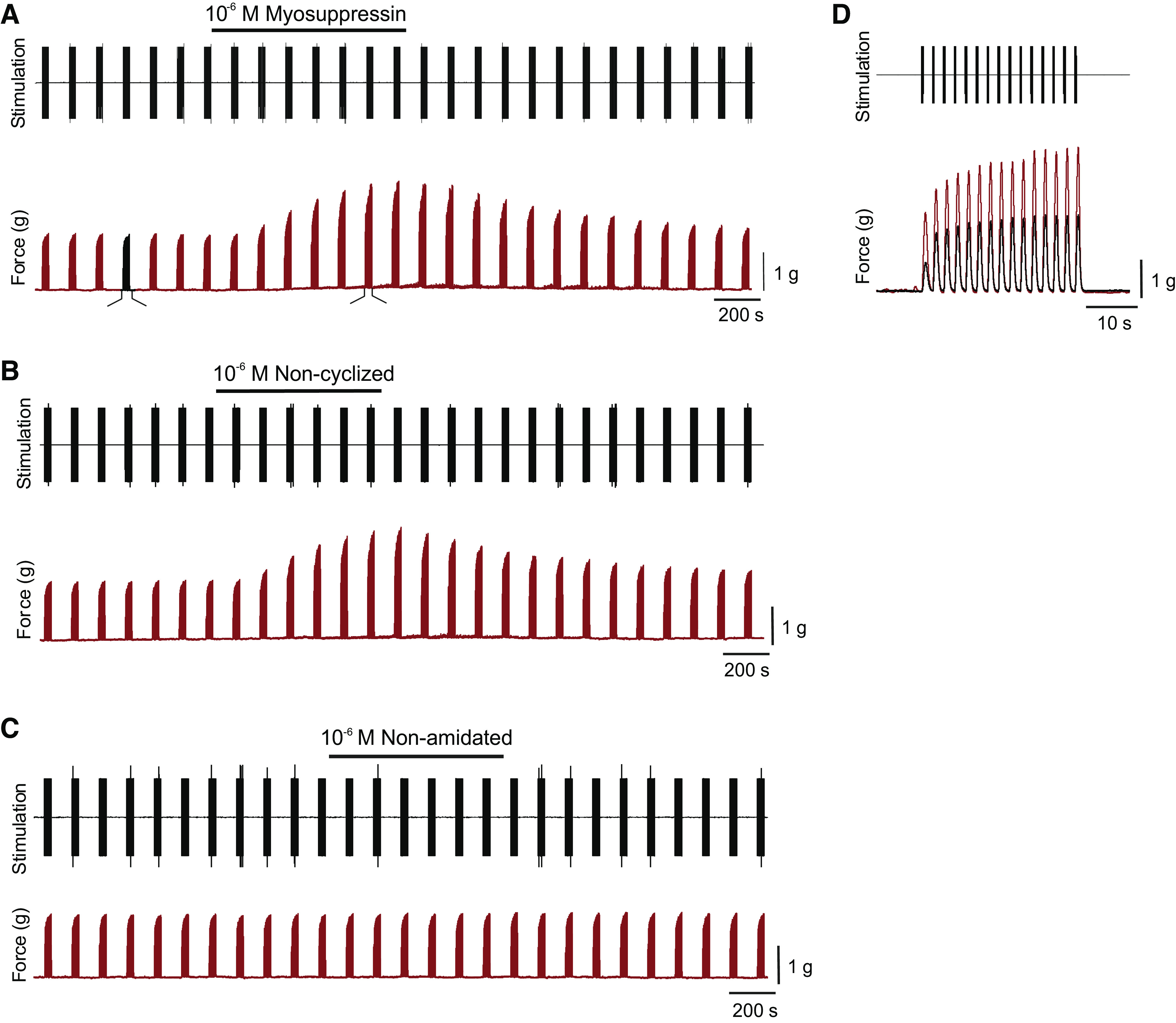
Nonamidated myosuppressin does not induce increased cardiac contraction force upon stimulation. Stimulated preparation recordings during application of 10^−6^ M full (*A*), noncyclized (*B*), and nonamidated (*C*) myosuppressin. The recordings shown from a single individual are representative of effects observed across individuals. *D*: comparison of a single bout of bursts from recording in *A* indicated by brackets underneath recording in control condition (black) and during the application of myosuppressin (red). Myosuppressin elicited a significant increase in all 15 stimulated contractions of this bout of bursts. Bars above traces represent time of peptide application.

Stimulated muscle contractions were used to ask whether each isoform of myosuppressin had an effect on the periphery of the cardiac neuromuscular system (Fig. 4). Given that the increase caused by myosuppressin in whole heart preparations is at least partially mediated by actions at the level of the muscle/neuromuscular junction, we predicted that myosuppressin and noncyclized myosuppressin would elicit increases in the amplitude of stimulated contractions. In contrast, nonamidated myosuppressin did not cause any increase in contraction amplitude in whole heart preparations; thus, we predicted that it would not cause any change in the amplitude of stimulated contractions.

**Figure 5.**
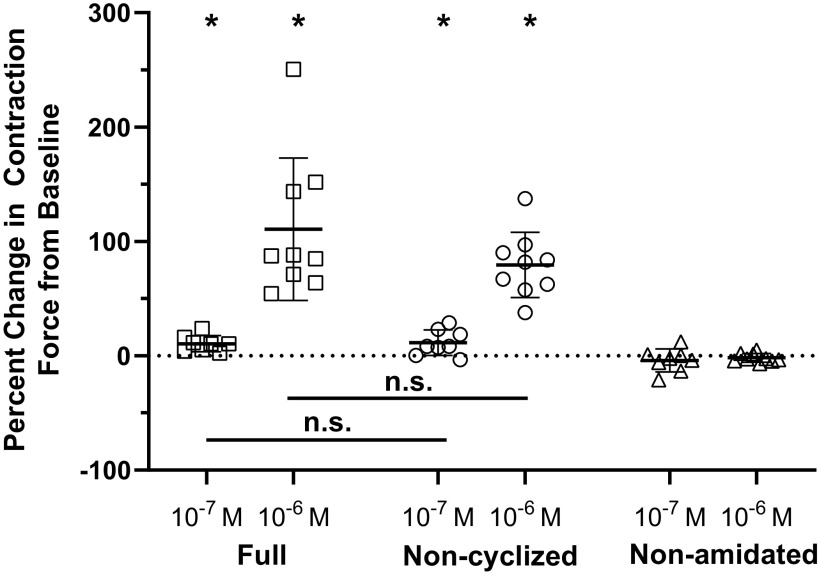
Myosuppressin and noncyclized myosuppressin cause similar changes in the force of stimulated contractions, whereas nonamidated myosuppressin does not alter contraction force. Graph shows percent change from baseline for force of the average of the 14th and 15th contraction in each successive bout during the applications of 10^−6^ M (*n* = 9) and 10^−7^ M (*n* = 8) full, noncyclized, and nonamidated myosuppressin. *Significant change from baseline [one sample *t* test, *P* < 0.05, *n* = 9 (for 10^−6^ M) or *n* = 8 (for 10^−7^ M)]. Error bars represent SD. The three means differ significantly from each other at both concentrations (one-way ANOVA, *P* = 0.0059, *n* = 8 at 10^−7^ M, *P* < 0.0001, *n* = 9 at 10^−6^ M). Full and noncyclized myosuppressin cause changes that are different from nonamidated myosuppressin (Tukey’s multiple comparisons: full 10^−7^ M, *P* = 0.016; noncyclized 10^−7^ M, *P* = 0.0105; full 10^−6^ M, *P* < 0.0001; noncyclized 10^−6^ M *P* = 0.0006), but are not different from each other (Tukey’s multiple comparisons: 10^−7^ M, *P* = 0.98; 10^−6^ M, *P* = 0.24).

Consistent with these predictions, application of both myosuppressin and noncyclized myosuppressin (10^−6^ M) caused a significant increase in contraction amplitude relative to baseline (Fig. 5, one sample *t* test, *P* = 0.0007 for myosuppressin and *P* < 0.0001 for noncyclized myosuppressin, *n* = 9). Contraction amplitude continued to increase through the early part of the wash period, suggesting long-lasting effects of the peptides. In contrast, nonamidated myosuppressin did not cause a significant change in the amplitude of contractions (Fig. 5, one sample *t* test, *P* = 0.21, *n* = 9). As in the whole heart, the effects of myosuppressin and noncyclized myosuppressin did not differ significantly from one another (Fig. 5, Tukey’s multiple comparisons, *P* = 0.24, *n* = 9). Similar effects were observed at 10^−7^ M; however, they were less pronounced than at the higher concentration.

### Myosuppressin Receptors Are Expressed in the Cardiac Muscle

**Figure 6.**
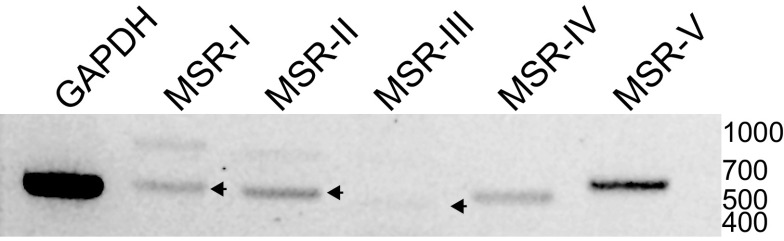
PCR-based confirmation of *HaMSR* expression in cardiac muscle. Representative image from eight biological replicates of *HaMSR I-V* expression in cardiac muscle. Arrowheads indicate products of expected sizes; all products were sequence validated. cDNA quality was confirmed by amplification of a 500-bp fragment of *HaGAPDH*.

All of the full-length *H. americanus* MSRs previously characterized (20) have structural features consistent with serpentine seven transmembrane domain G-protein-coupled receptors (GPCRs) (Pfam family PF10324) and exhibit 30%–35% amino acid identity with *Drosophila* receptors functionally characterized as MSRs. Given sequence similarities, it is likely that the HaMSRs couple through a Gα_i/o_ cAMP inhibition pathway, as reported for the silkmoth MSR (25). Given the presence of the five HaMSR transcripts in lobster transcriptomic datasets and potential differential expression in cardiac ganglia, we examined the expression of the MSRs in cardiac muscle cDNA across eight biological replicates. Sequence validated amplicons of the expected sizes for all five HaMSRs were identified in the cardiac muscle (Fig. 6), albeit not consistently across replicates (Table 2), suggesting possible conditional regulation of transcription and/or limited transcript abundance.

**Table 2.** Number of biological replicates in which an amplicon of the expected size was present in cardiac muscle cDNA samples

Transcript	Amplicon Present
MSR-I	6/8
MSR-II	6/8
MSR-III	8/8
MSR-IV	7/8
MSR-V	6/8
GAPDH	8/8

### All Myosuppressin Isoforms Alter the Ganglionic Bursting Pattern

**Figure 7.**
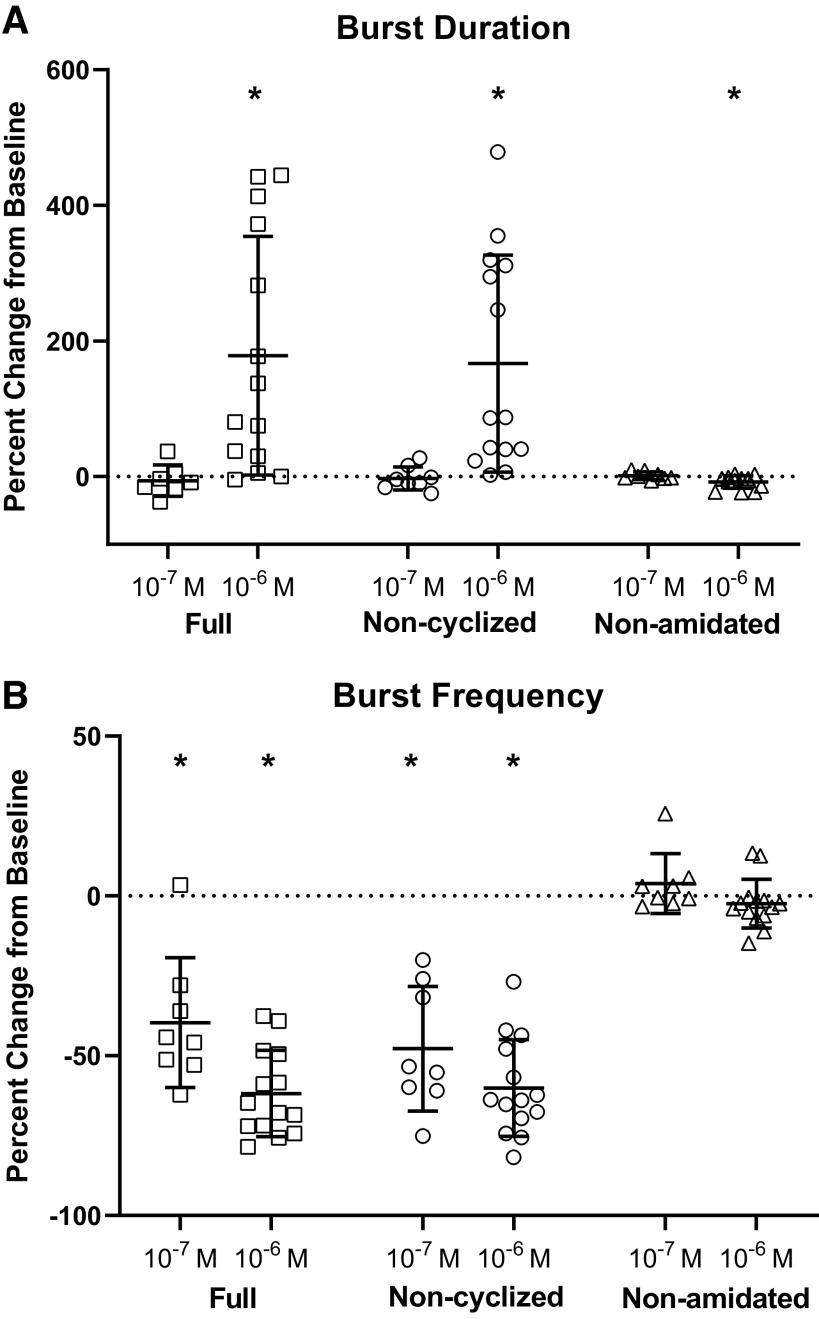
Myosuppressin and noncyclized myosuppressin alter burst characteristics in the isolated, intact cardiac ganglion in a dose-dependent manner. Myosuppressin isoforms elicited changes in burst duration (*A*) and burst frequency (*B*) at 10^−6^ M (*n* = 14), but only altered burst frequency at 10^−7^ M (*n* = 8). *Significant change from baseline [one sample *t* tests: burst duration (full, 10^−6^ M, *P* = 0.0023; noncyclized, 10^−6^ M, *P* = 0.0018; nonamidated, 10^−6^ M, *P* = 0.008), burst frequency (full, 10^−7^ M, *P* = 0.0009; full, 10^−6^ M, *P* < 0.0001; noncyclized, 10^−7^ M, *P* = 0.0002; noncyclized, 10^−6^ M, *P* < 0.0001)]. One-way ANOVA, error bars indicate SD. Changes in burst duration were larger in magnitude in response to 10^−6^ M full and noncyclized myosuppressin than in response to 10^−7^ M concentrations [Mann–Whitney tests: burst duration (full, *P* = 0.0008; noncyclized, *P* = 0.0001). Changes elicited by the isoforms in burst duration at 10^−6^ M concentrations and changes in burst frequency at both concentrations differ significantly from each other (one-way ANOVAs, *P* < 0.0001). Full and noncyclized myosuppressin elicited increases in burst duration and decreases in burst frequency that were significantly larger in magnitude than those elicited by non-amidated myosuppressin.

Application of each isoform to the isolated cardiac ganglion (ICG) resulted in observable changes in burst characteristics, consistent with previous reports of mature myosuppressin application (17). To quantify the effects of myosuppressin isoforms, burst characteristics, including burst duration and burst frequency, were measured at the time of peak response to the peptide. To compare CGs with different baseline bursting patterns, data were normalized by comparison of percent change from baseline. When superfused over the intact CG, application of myosuppressin and noncyclized myosuppressin elicited large decreases in the burst frequency of the CG at both 10^−6^ M and 10^−7^ M, but an increase in the duration of bursts was observed only at 10^−6^ M (Fig. 7, *A* and *B*). The changes elicited by these two peptides did not differ significantly from one another for either concentration applied to the CG (Tukey’s multiple comparisons: 10^−6^ M, *P* = 0.91 and *P* = 0.96 for burst frequency and burst duration, respectively, *n* = 14; 10^−7^ M, *P* = 0.61 and *P* = 0.92 for burst frequency and burst duration, respectively, *n* = 8). These results were consistent with the effects of myosuppressin found in previous studies (17). Interestingly, in spite of the fact that the whole heart and isolated CG differ in the presence versus absence of feedback pathways, the effects elicited by the mature versus noncyclized myosuppressin on cycle frequency in these two preparations were not significantly different (Tukey’s multiple comparisons, *P* = 0.83 and *P* = 0.40 for myosuppressin and noncyclized myosuppressin, respectively, *n* = 13 for whole heart and *n* = 14 for ICG).

**Figure 8.**
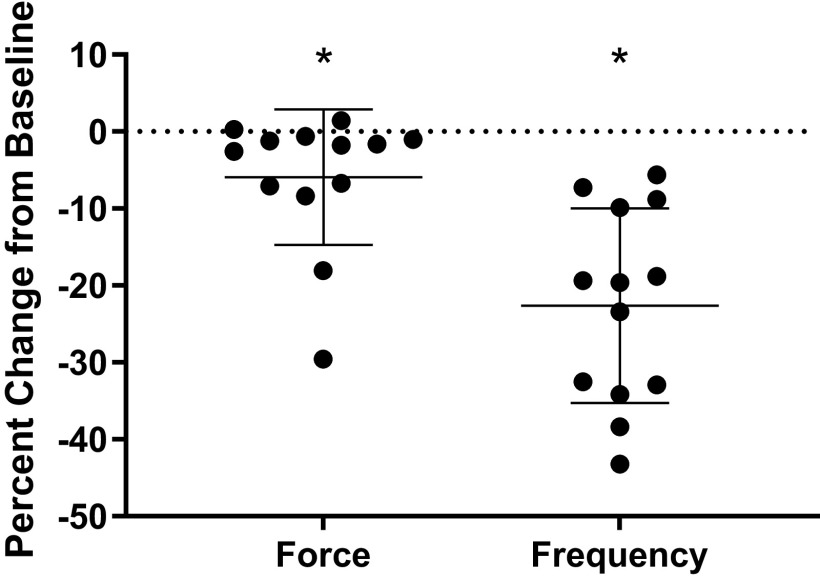
Extensive variability in both frequency and force was recorded in response to application of nonamidated myosuppressin in whole heart preparations. Replot of preparations (*n* = 13) exposed to 10^−6^ M concentrations of nonamidated myosuppressin from Fig. 3 to show variability. Graphs show percent change in baseline, with each point representing one individual preparation. With respect to frequency, in particular, some individuals responded to peptide with large decrease, while other preparations showed very little change from baseline conditions. *Significant change from baseline (one sample *t* tests, *P* < 0.05, *n* = 13). Changes elicited by nonamidated myosuppressin are variable [range (% change): amplitude, 37.60, frequency, 31.01]. Error bars represent SD.

In contrast, nonamidated myosuppressin did not significantly alter burst frequency (one sample *t* test, *P* = 0.26, *n* = 14) at either concentration, although it did significantly alter burst duration when applied at a 10^−6^ M concentration (Fig. 7*A*, one sample *t* test, *P* = 0.008). Surprisingly, this significant change in burst duration was a decrease, which was small in magnitude and contrasted with the large increase in burst duration elicited by both mature and noncyclized myosuppressin (Fig. 7*A*). Although the mean decrease in frequency elicited by nonamidated myosuppressin in whole heart preparations was small, responses differed considerably from animal to animal (Fig. 8). Given the marked variability in response to nonamidated myosuppressin application to the whole heart and the differential action of the peptide on the intact ganglion, we first asked whether ganglia taken from whole hearts that showed relatively robust responses to nonamidated myosuppressin were more responsive to this isoform than were CGs isolated from relatively unresponsive whole hearts. Second, because recent studies (20) have shown that the premotor and motor neurons of the CG respond differentially to mature myosuppressin, we asked whether either of these groups of neurons would respond to the nonamidated isoform when physically decoupled. If the nonamidated myosuppressin acts more strongly on one of the two neuron types, it is possible that this effect may be masked in the intact ganglion. We thus used two experimental preparations/protocols to further assess variability: *1*) a paired whole heart and ICG preparation to examine the extent of modulation within an individual, and *2*) application of nonamidated myosuppressin to the physically decoupled premotor and motor neurons to assess whether the peptide acts on one or both neuron types.

### Response to Nonamidated Myosuppressin Is Variable across Individuals

**Figure 9.**
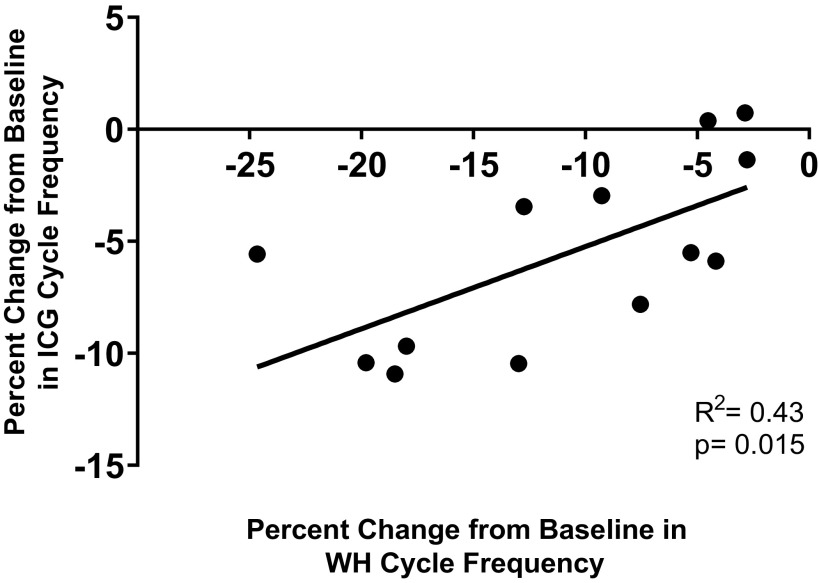
The frequency of contractions in the whole heart (WH) and the frequency of bursts in the isolated cardiac ganglion (ICG) from the same individual in response to nonamidated myosuppressin are correlated. Nonamidated myosuppressin (10^−6^ M) elicited changes in the whole heart contraction frequency that were correlated with the changes observed in the burst frequency of the ganglion when isolated from the same individual (*R*^2^ = 0.43, *P* = 0.015, *n* = 13). Line represents the linear regression of the data set.

Application of nonamidated myosuppressin to the whole heart and the ganglion of the same individual enabled us to assess variable levels of susceptibility across individuals. When nonamidated myosuppressin was perfused through the whole heart and superfused over the isolated ganglion taken from the same heart at 10^−6^ M, there was a moderate correlation between the extent of modulation of frequency in these two preparations (Fig. 9, *R*^2^ = 0.43, *P* = 0.015, *n* = 13).

**Figure 10.**
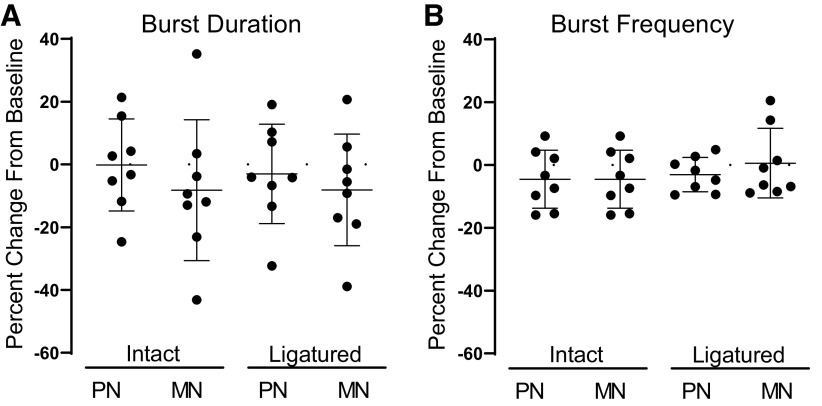
Nonamidated myosuppressin elicits highly variable responses from the neurons of the cardiac ganglion. Application of nonamidated myosuppressin (10^−6^ M) did not elicit significant changes from baseline in either the burst duration (*A*) or burst frequency (*B*) of the intact and ligatured premotor (PN) and motor (MN) neurons (*n* = 8). However, the changes elicited by the peptide were highly variable among preparations in both burst duration [range (% change): PN, int, 45.99; MN, int, 51.43; PN, lig, 78.35; MN, lig, 59.56) and burst frequency [range (% change): PN, int, 25.13; MN, int, 14.37; PN, lig, 25.13 MN, 29.39]. This was the case for both neuron types before as well as after ligature. Error bars indicate SD.

In a new subset of individuals, when nonamidated myosuppressin was superfused over intact ganglia, and again after the tightening of a ligature to physically decouple the premotor neurons (PN) and motor neurons (MN), no significant changes in burst characteristics were observed in either neuron type (Fig. 10, *n* = 8). These data differ from the significant decrease in burst duration observed in the population of intact ganglia superfused with all myosuppressin isoforms previously (Fig. 7, *n* = 14). However, assessment of the responses to nonamidated myosuppressin in the isolated, intact, and ligatured neurons here reveals that nonamidated myosuppressin elicited highly variable changes in burst duration [Fig. 10*A*, range (% change): PN, int, 45.99; MN, int, 51.43; PN, lig, 78.35; MN, lig, 59.56] and burst frequency [Fig. 10*B*, range (% change): PN, int, 25.13; MN, int, 14.37; PN, lig, 25.13 MN, 29.39], suggesting that population-wide assessment of modulation by nonamidated myosuppressin may be less meaningful than for the other isoforms.

## DISCUSSION

Post-translational modifications are essential mechanisms that diversify peptide function and enable dynamic signaling capacity in complex networks. These structural alterations allow for robust regulation of various cellular functions, including signaling pathways. Previous studies of the physiological effects of neuromodulators on crustacean CPGs have taken for granted the necessity of PTMs for bioactivity. Here, we show that certain PTMs affect the bioactivity of a well-characterized neuropeptide, myosuppressin, in the cardiac neuromuscular system of the lobster. Myosuppressin is present in the neurons of the cardiac ganglion (20) and the eyestalk ganglia (24), suggesting that this peptide is both hormonally released from the sinus gland and locally released from the neurons themselves, consistent with the range of peptide concentrations applied here.

Removing cyclization of the N-terminal glutamine did not change the effects of myosuppressin at the neural level, at the level of the neuromuscular junction/muscle, or in the intact whole heart system. In all preparations tested, the magnitude of change induced by application of noncyclized myosuppressin was not significantly different from the fully modified peptide, suggesting that removal of the cyclization does not affect the modulatory capabilities of myosuppressin. Previous work in various species has shown that the cyclization of glutamine/glutamic acid residues often functions to stabilize the peptide and prevent it from degrading before reaching target cells (e.g., Refs. 26 and 27). For example, when the pyroglutamate on the related leucomyosuppressin in *Leucophaea maderae* cockroaches was removed, the peptide retained similar bioactivity (28), suggesting that the removal of this residue does not alter the properties of the peptide itself, but may instead prevent degradation before reaching target cells. Thus, given the manner in which myosuppressin isoforms were applied to isolated portions of the cardiac neuromuscular system here, antidegradative properties conferred by the N-terminal cyclization could not be observed and the removal of the N-terminal cyclization of myosuppressin did not alter the experimental effects of myosuppressin.

Removal of the amidation signal and replacement with a glycine residue (i.e., the sequence predicted to exist before amidation) on the myosuppressin C-terminus substantially decreased the effects of the peptide. The nonamidated form of myosuppressin is an intermediate form of the full peptide before N-terminal amidation, thus, it is possible, although not confirmed, that the nonamidated form of the peptide exists in the hemolymph. Although applications of nonamidated myosuppressin did elicit changes to both the frequency and amplitude of whole heart contractions, these changes were significantly smaller than those elicited by the other isoforms. Similarly, in the adult heart of *Drosophila melanogaster*, the effect of the free acid analog of *Drosophila* myosuppressin is reduced relative to that of the native myosuppressin (29). Unlike the other two isoforms, nonamidated myosuppressin did not elicit a bi-phasic response to myosuppressin; specifically, there was no evidence of a secondary increase in contraction amplitude. The decreased frequency of whole heart contractions in the presence of nonamidated myosuppressin suggests that this peptide isoform acts on the CG and decreases the burst frequency. If this isoform of the peptide was unable to exert effects at the periphery, a decrease in burst frequency would cause a decrease in contraction amplitude due to the nonlinear translation of bursting parameters into contractions via the neuromuscular transform (18). We thus predicted that nonamidated myosuppressin would exert effects on the ICG, but not at the periphery.

We examined the responses of both the periphery (neuromuscular junction/muscle) and the CG itself. Consistent with our predictions, nonamidated myosuppressin has no effect at the periphery. Surprisingly, however, it likewise did not elicit a decrease in burst frequency when applied to the ICG. It did, however, elicit a small decrease in burst duration, something not seen in response to mature myosuppressin (17), in one set of experiments. Although these changes could explain the decrease in contraction amplitude recorded in whole hearts, they cannot explain the decrease in contraction frequency. Interestingly, in a separate set of experiments on ganglia that were physically ligatured to decouple the premotor and motor neurons, there were no significant changes in the bursting pattern in either neuron type in the intact or ligatured state. Given that the response to nonamidated myosuppressin at the neural level was highly variable, it is possible that significant modulation of individual ganglia was not captured when assessed across individuals. The effect of nonamidated myosuppressin on the premotor and motor neurons in the intact ganglion may be due to the cumulative, small effects of the peptide on both cell types and/or the interaction between the neuron types. Comparison between the extent of modulation of the whole heart and the isolated ganglion in the same individual in response to nonamidated myosuppressin revealed a significant correlation between the extent of central (i.e., intact ganglion) and global (i.e., whole heart) modulation that was variable across individuals. Together, these data suggest that mature and noncyclized myosuppressin exert their effects by acting both on the cardiac ganglion and at the periphery, while the nonamidated isoform is able to exert effects only at the level of the ganglion, driving small but significant changes in the whole heart (Table 3). Moreover, these central effects are much smaller than those of the other isoforms. This in turn suggests the possibility of differential distribution of myosuppressin receptors, coupled with differential affinity for these isoforms.

**Table 3.** Summary of effects of myosuppressin isoforms on the cardiac neuromuscular system

	Full Myosuppressin	Non-Cyclized Myosuppressin	Non-Amidated Myosuppressin
Whole heart			
Frequency	Large ↓	Large ↓	Small ↓
Amplitude	Biphasic, small ↓ then large ↑	Biphasic, small ↓ then large ↑	Small ↓
Cardiac muscle			
Force	↑	↑	No change
Intact cardiac ganglion			
Burst duration	↑	↑	Small ↓
Burst frequency	↓	↓	No change
Isolated motor neurons			
Burst duration			No change
Burst frequency			No change
Isolated premotor neurons			
Burst duration			No change
Burst frequency			No change

Arrows indicate direction of changes: ↑, increase; ↓, decrease.

Oleisky et al. (20) demonstrated differential expression of the five myosuppressin receptors (MSRs) in the premotor and motor neurons of the cardiac ganglion; the neurons of the ganglion expressed only MSRII, III, and IV. Here, we show that all five MSRs are present in the *Homarus* cardiac muscle. Given that nonamidated myosuppressin does not appear to act on the neuromuscular junction to elicit the observed changes in the whole heart, one possibility to explain the changes elicited by nonamidated myosuppressin in the whole heart and the CG may be a differential binding capability of nonamidated myosuppressin to the MSRs. It is possible that the amidation alters the binding capacity of the peptide to different receptor variants, and that an MSR present at higher levels in the neurons of the CG, but lower levels in the cardiac muscle, mediates the effects of nonamidated myosuppressin in the ganglion. In addition, MSRs not expressed in the CG but expressed in the muscle (i.e., MSR I, V) could potentially mediate the response to full and noncyclized myosuppressin observed in cardiac muscle. To resolve this issue, we would require experiments examining the binding properties of all five receptors to the amidated and nonamidated peptides, which is beyond the scope of this study.

For many peptides, amidation has been shown to be necessary for bioactivity (30, 31); thus, it is unsurprising that nonamidated myosuppressin elicited distinct changes in comparison to other myosuppressin isoforms. However, given the capacity of nonamidated myosuppressin to elicit significant changes in a subset of individuals, our data suggest that the amidation signal may not be required for modulation, and that nonamidated myosuppressin may have differential affinity for myosuppressin receptors that allow for significant changes in patterned output in some individuals. More broadly, these data suggest that there exist peptide receptors that do not require an amidation signal for partial binding and signaling activity. However, we cannot ignore the possibility that the presence of a glycine residue may have altered peptide binding capability. Other crustacean peptides identified with free G-acid residues, such as the nonamidated peptide tested here, exert no physiological effects (19), which raises the possibility that the presence of the glycine residue on nonamidated myosuppressin may have altered its activity on the cardiac system. In contrast, adding or removing a C-terminal amide to members of at least one other crustacean peptide family, the C-type allatostatins, does not alter the physiological response of the *H. americanus* heart to the peptide (unpublished observations), suggesting that the importance of this PTM may depend on other aspects of the peptide’s structure.

In the course of this study, we replicated previous experiments (18, 20). Our experiments were consistent when using locally released (i.e., 10^−6^ M) peptide concentrations (18, 20), but there were some differences at hormonal concentrations (i.e., 10^−7^ M), specifically in the burst duration of the CG. We did not see an increase in burst duration in the intact ganglion in response to 10^−7^ M myosuppressin, which differs from the increase seen in Stevens et al. (17), but is consistent with Oleisky et al. (20), suggesting that there may be differences in the state of lobster across populations and years.

Taken together, this study provides evidence that PTMs affect the bioactivity of peptide neuromodulators. For many peptides, amidation is thought to be necessary to bind and elicit significant changes within target tissues. Here, we show that removing the amidation signal from the peptide altered the effects of the peptide but did not render it completely nonfunctional. Nonamidated myosuppressin was found to have significant effects on the intact cardiac neuromuscular system, but limited, variable effects on the isolated nervous system and no effect on the periphery of the system. Due to the differential distribution of peptide receptors across the system, these data raise the possibility that the amidation signal is crucial for binding to a subset of peptide receptors. The ability of differential peptide structure to increase the functional flexibility of pattern-generating systems provides insight regarding the role of post-translational modifications in peptidergic modulation on signaling networks. Moreover, this emphasizes the role of amidation versus cyclization as a key factor in maintaining functionality of this neuropeptide.

## GRANTS

This research was funded by National Science Foundation Grant IOS-1354567, the National Institutes of Health Award 8 P20 GM103423 from National Institute of General Medical Sciences (NIGMS), the Doherty Charitable Foundation, and the Arnold and Mabel Beckman Foundation.

## DISCLOSURES

No conflicts of interest, financial or otherwise, are declared by the authors.

## AUTHOR CONTRIBUTIONS

E.R.O., M.E.S., and P.S.D. conceived and designed research; E.R.O., M.E.S., J.J.H., and P.S.D. performed experiments; E.R.O., M.E.S., J.J.H., and P.S.D. analyzed data; E.R.O., M.E.S., J.J.H., and P.S.D. interpreted results of experiments; E.R.O., M.E.S., J.J.H., and P.S.D. prepared figures; E.R.O., M.E.S., and P.S.D. drafted manuscript; E.R.O., M.E.S., J.J.H., and P.S.D. edited and revised manuscript; E.R.O., M.E.S., J.J.H., and P.S.D. approved final version of manuscript.
